# Supplementary material for: Spatial distribution, pollution, and health risk assessment of heavy metal in agricultural surface soil for the Guangzhou-Foshan urban zone, South China
Source: PLoS One. 2020 Oct 8;15(10):e0239563. doi: 10.1371/journal.pone.0239563 (PMC7544098; doi:10.1371/journal.pone.0239563)
Supplement: S1 File — (DOCX) [file pone.0239563.s001.docx]

**Spatial distribution, pollution, and health risk assessment of heavy metal in agricultural surface soil for the Guangzhou-Foshan urban zone, South China**

Yi Xiao^1^, Mingyan Guo^1^, Xiaohong Li^1^, Xixiang Luo^1^, Ruikang Pan^1^, Tingping Ouyang^1, 2^^[[1]](#footnote-1)^*

^1^ School of Geography, South China Normal University, Guangzhou 510631, China

^2^ Key Laboratory of Ocean and Marginal Sea Geology, Guangzhou Institute of Geochemistry, Chinese Academy of Sciences, Guangzhou 510640, China

**The formulas of human health risk assessment.**

For non-carcinogenic risk, the average daily intake (ADD) of heavy metals of each exposure pathway was calculated using Eqs. (1-4). The total non-carcinogenic risk (THI) is a sum of hazard quotient (HQ) (Eq. (6)), which was estimated by dividing the ADD by the corresponding reference dose (RfD) of all assessed heavy metals through all exposure pathways (Eq. (5)). As for the carcinogenic risk (CR), lifetime average potential daily dose (LADD) was calculated for each exposure pathway using Eqs. (7-10). Subsequently, CR for each heavy metal and total CR (TCR) was calculated using Equations (11) and (12), respectively.

${ADD}_{cons}=\frac{C\times TF\times{IR}_{p}\times EF\times ED}{BW\times AT}\times{10}^{-6}$ (1)

${ADD}_{ing}=\frac{C\times{IR}_{s}\times EF\times ED}{BW\times AT}\times{10}^{-6}$ (2)

${ADD}_{dermal}=\frac{C\times SA\times AF\times ABS\times EF\times ED}{BW\times AT}$ (3)

${ADD}_{inh}=\frac{C\times{IR}_{i}\times EF\times ED}{PEF\times BW\times AT}$ (4)

$HQ=\sum\frac{ADD}{Rfd}$ (5)

$THI=\sum HQ$ (6)

$L{ADD}_{cons}=\frac{C\times TF\times{IR}_{p}\times EF\times ED}{BW\times LT}\times{10}^{-6}$ (7)

$L{ADD}_{ing}=\frac{C\times{IR}_{s}\times EF\times ED}{BW\times LT}\times{10}^{-6}$ (8)

${LADD}_{dermal}=\frac{C\times SA\times AF\times ABS\times EF\times ED}{BW\times LT}$ (9)

$L{ADD}_{inh}=\frac{C\times{IR}_{i}\times EF\times ED}{PEF\times BW\times LT}$ (10)

$CR=\sum(LADD\times SF)$ (11)

$TCR=\sum{CR}$ (12)

where, ${ADD}_{cons}$, ${ADD}_{ing}$,${ADD}_{dermal}$, and ${ADD}_{inh}$ represent average daily exposure dose (mg/kg/day) through pathways products consumption, soil ingestion, dermal contact, and air inhalation, respectively. Similarly, $L{ADD}_{cons}$, $L{ADD}_{ing}$, ${LADD}_{dermal}$, and $L{ADD}_{inh}$ represent daily carcinogenic risk exposure dose (mg/kg/day) through the mentioned exposure pathways, respectively.${IR}_{s}$and ${IR}_{p}$ are the uptake rates of soil and agricultural products (mg/day), respectively. TF is the soil-plant transfer factor;${IR}_{i}$ is the soil suction rate (m^3^/day). EF is the contact frequency (day/year); ED is the duration of exposure (years); BW is the body weight (kg); AT is the time period of average dose (days); PEF is the emission factor (m^3^/kg); SA is skin exposure surface area (cm^2^); AF is the adhesion factor (kg/cm^2^/day). ABS is a skin absorption factor; RfD is the reference dose (mg/kg/day). SF is carcinogenic factor (mg/kg/day). LT is the average life (days). Among the mentioned parameters, Values of RfD, SF, and TF used in the present study are listed in supplementary S1 Table for every element. The values of the other parameters used in the present study are listed in supplementary S2 Table for children (aged 0-17) and adults (18 and over 18), respectively.

**Table S1. Reference dose (RfD), slope factor (SF) and transfer factor (TF) of heavy metals for health risk assessment.**

| Metals | RfD(mg/kg·day) | | | SF | | | TF^1^ | | References |
| --- | --- | --- | --- | --- | --- | --- | --- | --- | --- |
|  |  |  |  |  |  |  |  |  |  |
|  | ingestion | dermal | inhalation | ingestion | dermal | inhalation | soil-rice | soil-vegetables |  |
| Cd | 1.0E-03 | 2.5E-05 | 5.71E-05 | 6.1 | 6.1 | 6.3 | 0.08 | 0.079 | (1-3) |
| Pb | 3.5E-03 | 5.25E-04 | 3.52E-03 | - | - | - | 0.0017 | 0.002 | (1, 3) |
| Cu | 0.04 | 0.012 | 0.0402 | - | - | - | 0.081 | 0.04 |  |
| Cr | 0.003 | 6.0E-05 | 2.86E-05 | 0.5 | 20 | 42 | 0.0084 | 0.0069 | (1-3) |
| Zn | 0.3 | 0.06 | 0.3 | - | - | - | 0.11 | 0.045 | (1, 3) |
| Ni | 0.02 | 0.0008 | 0.026 | - | - | - | 0.018 | 0.005 | (2, 4) |
| As | 3.0E-04 | 3.0E-04 | 3.0E-04 | 1.5 | 3.66 | 15.1 | 0.002 | 0.001 | (2, 4) |
| Hg | 1.6E-04 | 1.6E-04 | 8.57E-05 | - | - | - | 0.027 | 0.011 | (1, 2) |

Notes: ^1^ Transfer factor defined as the ratio of metal concentration in plant to that in soil. As rice and vegetables are the main food crops in Guangzhou and Foshan (Zhang et al., 2018), the intake of rice and vegetables is mainly considered as the consumption of agricultural products.

**Table S2. Parameters used for the health risk assessment in this study.**

| Parameters | Description | Units | Value | References |
| --- | --- | --- | --- | --- |
| ${IR}_{s}$ | ingestion rate of soil | mg/day | 20 for adults and 50 for children | (5, 6) |
| ${IR}_{p}$ ^1^ | including the ingestion rate of rice and vegetable | Mg/day | for rice, 302000 to adults and 257000 to children; for vegetables, 24300 for adults and 23800 for children | (7, 8) |
|  |  |  |  |  |
| $EF$ | exposure frequency | day/year | 350 | (9) |
| $ED$ | exposure duration | year | 24 for adults and 6 for children | (6) |
| $BW$ | average body weight | kg | 57 for adults and 24.7 for children | (7, 10) |
| $AT$ | average exposure time | days | ED×365 | (11) |
| $SA$ | surface area of skin | cm^2^ | 5700 for adults and 2800 for children | (11) |
| $AF$ | skin adherence factor | kg/cm^2^-day | 2 × 10^-7^ for adults and 10^-6^ for children | (6, 12） |
| $ABS$ | dermal absorption factor | unitless | 0.001 | (13) |
| ${IR}_{i}$ | inhalation rate | m^3^/day | 16 for adults and 7.6 for children | (14, 15) |
| $PEF$ | emission factor | m^3^/kg | 1.36×10^9^ | (16) |
| $LT$ | Life time expressed in day | day | 76.49 × 365 | (10) |

Notes: ^1^ ${IR}_{p}$is the ingestion rates of rice and vegetables. As rice and vegetables are the main food crops in Guangzhou and Foshan, the intake of rice and vegetables is mainly considered as agricultural products. The recommended intake values of staple food, vegetables and fruits for adults and children in Guangdong Province are obtained in the Chinese population exposure manual (7, 8). In order to ensure the consistency between the units used for the intake rate and those used for the concentration data (i.e. if the concentration of heavy metals is measured by the dry weight of fruits and vegetables, then the dry weight units should be used for the intake value), the intake of wet-weight is converted to dry-weight intake using the percentages of water content shown in the table of Chinese food ingredients and the following equation:

$${IR}_{dw}={IR}_{ww}\left\lfloor\frac{100-W}{W} \right\rfloor$$

Where, ${IR}_{dw}$ = dry-weight intake rate,$\mathrm{IR}_{\mathrm{ww}}$ = wet-weight intake rate, and W = percent water content.

**References**

1. Cao HB, Chen JJ, Zhang J, Zhang H, Qiao L, Men Y. Heavy metals in rice and garden vegetables and their potential health risks to inhabitants in the vicinity of an industrial zone in Jiangsu, China. J. Environ. Sci. , 2010. https://doi.org/10.1016/S1001-0742(09)60321-1.

2. Ma WC, Tai LY, Qiao Z, Zhong L, Wang Z, Fu KX et al. Contamination source apportionment and health risk assessment of heavy metals in soil around municipal solid waste incinerator: A case study in North China. Sci. Total Environ. 2018;631-632:348-57. Epub 2018/03/12. https://doi.org/10.1016/j.scitotenv.2018.03.011.

3. Zhang R, Chen T, Zhang Y, Hou YH, Chang QR. Health risk assessment of heavy metals in agricultural soils and identification of main influencing factors in a typical industrial park in northwest China. Chemosphere. 2020;252:126591. Epub 2020/04/03. https://doi.org/10.1016/j.chemosphere.2020.126591.

4. Zhang JR, Li HZ, Zhou YZ, Dou L, Cai LM, Mo LP, et al. Bioavailability and soil-to-crop transfer of heavy metals in farmland soils: A case study in the Pearl River Delta, South China. Environ. Pollut. 2018;235:710-9. Epub 2018/01/18. https://doi.org/10.1016/j.envpol.2017.12.106.

5. Jiang YX, Zeng XC, Fan XT, Chao SH, Zhu ML, Cao HB. Levels of arsenic pollution in daily foodstuffs and soils and its associated human health risk in a town in Jiangsu Province, China. Ecotoxicol. Environ. Saf. 2015;122:198-204. Epub 2015/08/11. https://doi.org/10.1016/j.ecoenv.2015.07.018.

6. US EPA. Exposure Factors Handbook 2011 Edition (Final). U.S. Environmental Protection Agency, Washington, DC, EPA/600/R-09/052F. 2011.

7. Duan XL. Highlights of the Chinese Exposure Factors Handbook (children) China Environment press, Beijing, China. (in Chinese).2016.

8. MEP. Ministry of Environmental Protection of the People’s Republic of China, Exposure Factors Handbook of Chinese Population (Adults). China Environmental Science Press, Beijing(in Chinese). 2013.

9. US EPA. Risk Assessment Guidance for Superfund: Volume I: Human Health Evaluation Manual (Part A). Office of Solid Waste and Emergency Response, US Environmental Protection Agency. 1989.

10. Duan XL, Zhao XG, Wang BB, Chen YT, Cao SZ. Highlights of the Chinese Exposure Factors Handbook (Adults). Science Press, Beijing, China. 2015.

11. US EPA. Risk Assessment Guidance for Superfund: Volume I: Human Health Evaluation Manual. Office of Superfund Remediation and Technology Innovation, Washington, DC, USA.2004.

12. Finley B, Proctor D, Scott P, Harrington N, Paustenbach D, Price P. Recommended Distributions for Exposure Factors Frequently Used in Health Risk Assessment. Risk Anal. 1994;14(4):533-53. https://doi.org/10.1111/j.1539-6924.1994.tb00269.x.

13. Ferreira-Baptista L, De Miguel E. Geochemistry and risk assessment of street dust in Luanda, Angola: A tropical urban environment. Atmos Environ. 2005;39(25):4501-12. https://doi.org/10.1016/j.atmosenv.2005.03.026.

14. Chabukdhara M, Nema AK. Heavy metals assessment in urban soil around industrial clusters in Ghaziabad, India: probabilistic health risk approach. Ecotoxicol Environ Saf. 2013;87:57-64. http://dx.doi.org/10.1016/j.ecoenv.2012.08.032

15. Jiang YX, Zeng XC, Fan XT, Chao SH, Zhu ML, Cao HB. Levels of arsenic pollution in daily foodstuffs and soils and its associated human health risk in a town in Jiangsu Province, China. Ecotoxicol. Environ. Saf. 2015;122:198-204. Epub 2015/08/11. https://doi.org/10.1016/j.ecoenv.2015.07.018.

16. US EPA. Supplemental Guidance for Developing Soil Screening Levels for Superfund Sites. Environmental Protection Agency, Washington, DC, USA. 2002.

1. *Corresponding author.

   E-mail addresses: oyangtp@m.scnu.edu.cn (Dr. Tingping Ouyang) [↑](#footnote-ref-1)
